# Effect of Dietary PUFAs and Antioxidants on Antioxidant and Anti-Inflammatory Functions of HDL in a Cohort of Women

**DOI:** 10.3390/antiox14101221

**Published:** 2025-10-10

**Authors:** Gianmarco Mola, Raffaella Riccetti, Domenico Sergi, Alessandro Trentini, Valentina Rosta, Angelina Passaro, Juana M. Sanz, Carlo Cervellati

**Affiliations:** 1 Department of Translational Medicine and for Romagna, University of Ferrara, Via Luigi Borsari 46, 44121 Ferrara, Italy; gianmarco.mola@unife.it (G.M.); domenico.sergi@unife.it (D.S.); psn@unife.it (A.P.); 2 Department of Environmental and Prevention Sciences, University of Ferrara, Via Luigi Borsari 46, 44121 Ferrara, Italy; raffaella.riccetti@unife.it (R.R.); alessandro.trentini@unife.it (A.T.); valentina.rosta@unife.it (V.R.); 3 Department of Chemical, Pharmaceutical and Agricultural Sciences, University of Ferrara, Via Luigi Borsari 46, 44121 Ferrara, Italy; szj@unife.it

**Keywords:** HDL, antioxidants, PUFAs, paraoxonase-1, myeloperoxidase

## Abstract

High-density lipoproteins (HDLs) protect against atherosclerosis through their antioxidant, anti-inflammatory, and other beneficial properties. Although interest is increasing in uncovering both physiological and external factors that influence these functions, definitive evidence remains lacking in this area. To fill this gap, we assessed for the first time how intake of saturated and unsaturated fatty acids and dietary antioxidants affects key HDL-associated proteins. We observed that myeloperoxidase (MPO) activity, a marker of HDL oxidation, was inversely correlated with total polyunsaturated fatty acids (PUFAs), omega-3 and omega-6 intake (*p* < 0.05), polyphenols (*p* < 0.001), and overall antioxidant capacity (*p* < 0.05). Levels of lipoprotein-associated phospholipase A2 also decreased with higher antioxidant consumption (*p* < 0.05). By contrast, glutathione peroxidase 3 (Gpx3) activity, a protective HDL enzyme, increased in tandem with omega-3 and antioxidant intake. Finally, a composite HDL-antioxidant/anti-inflammatory score integrating all measured proteins rose in association with total PUFAs (*p* < 0.001), omega-6 (*p* < 0.001), omega-3 (*p* < 0.01), polyphenols, and total antioxidants (*p* < 0.05). These findings suggest that higher dietary PUFA, especially omega-6, and antioxidant intake may enhance HDL’s atheroprotective properties.

## 1. Introduction

High-density lipoproteins (HDLs) are generally considered to be protective against atherosclerotic diseases [1,2]. However, current research shows that simply having a high concentration of HDL particles (HDL-p) or HDL cholesterol (HDL-C) does not automatically translate to anti-atherosclerotic benefits [3,4]. In fact, exceptionally high levels might not offer extra protection and could even be linked to increased risks of cardiovascular disease or overall mortality [1,2,3].

The protective effect of HDLs against atherosclerosis is dictated by their diverse biological functions, particularly their antioxidant anti-inflammatory properties [5,6,7]. These functions help counteract and alleviate the conditions that lead to early atheroma formation, such as endothelial dysfunction, low density lipoprotein (LDL) and HDL oxidation, and formation of foam cells [5,6,7,8]. These dynamic mechanisms, rather than HDL levels per se, play a key role in HDL-mediated cardiovascular benefits, as also demonstrated by a recent meta-analysis [9]. The functional efficiency of HDLs chiefly depends on the balance of the activities of several proteins with different proprieties [1,10,11,12,13]. These important constituents of HDL proteome include the main and most abundant apolipoprotein, APOA1, and a series of so-called “accessory proteins”, such as the protective and antioxidant Paraoxonase-1 and glutathione peroxidase 3 (GPX3), the pro-inflammatory lipoprotein-phospholipase A2 (Lp-PLA2), the pro-oxidant and pro-inflammatory myeloperoxidase (MPO) [1,6,7,10,11,12,14,15] and one of the main promoters of reverse cholesterol transport (which catalyzes the esterification of free cholesterol in the HDL surface, thereby promoting its biological maturation) with antioxidant activity [16], lecithin cholesterol acyltransferase (LCAT).

Despite increasing interest in this complex area, there remains a significant lack of literature concerning both endogenous and exogenous factors that can modulate the activity of individual HDL components or of the overall lipoprotein.

The study conducted by El Khoudary et al. highlights one of the potential endogenous factors affecting HDL functionality during the menopausal transition in women [17]. Their findings indicate that although HDL-C levels tend to rise in postmenopausal women, this increase is paradoxically associated with a decline in HDL’s overall functionality. According to the authors, this deterioration is primarily linked to the estrogen deficiency and shifts in body fat distribution that accompany menopause. These changes may help explain the observed relationship between rising HDL-C levels and the progression of atherosclerosis during this period [18].

Among the external influences on HDL function, diet emerges as a significant contributing factor. The most compelling evidence in this regard originates from in vitro studies exploring the potential effects of nutrients and food bioactives, particularly poly- and monounsaturated fatty acids (FAs, like PUFA and MUFA) and dietary antioxidants, in shaping HDL proteome and functionality [19,20,21,22,23]. Despite these preclinical findings being encouraging, the randomized controlled trials investigating the effects of dietary interventions on HDL proteome, especially on PON1, Lp-PLA2, and L-CAT were limited in numbers and reported inconsistent results [24,25]. While some studies reported that diets rich in antioxidants and PUFAs were associated with increased levels or activity of protective HDL-associated proteins [26,27], or with reductions in those potentially harmful [28,29], others failed to observe any significant effects [30,31]. A further limitation of the existing literature is the lack of studies evaluating the impact of these nutrients on the overall functionality of HDL.

Against this still inconclusive background, our study investigates the impact of menopause and dietary saturated and unsaturated FAs, as well as antioxidants, on HDL functionality. Particular attention is given to its antioxidant and anti-inflammatory properties, which we have attempted to integrate into a single composite score.

## 2. Materials and Methods

### 2.1. Subjects

The study involved 145 women recruited from the Menopause and Osteoporosis center at the University of Ferrara, Italy. The primary objective of this study was to investigate the potential influence of menopausal status on various metabolic, oxidative, and inflammatory biomarkers. A secondary aim was to assess the relationship between these changes and the participants’ nutritional habits. It was meticulously designed and carried out in line with the ethical principles outlined in the World Medical Association’s Declaration of Helsinki. The ethics committee of the University Hospital of Ferrara granted approval for the study on 20 May 2019 (reference: 207/2019/Sper/UniFe). Each participant gave written informed consent before being included in the study. According to the study inclusion criteria, both fertile and postmenopausal women were recruited for the study. Menopause was defined as at least 12 consecutive months of amenorrhea, as per the recent ReSTAGE modification of the STRAW staging criteria [32].

Individuals with any chronic diseases including diabetes, thromboembolism, neurodegenerative conditions, major systemic infections, autoimmune disorders, cancer, or kidney failure were excluded from the study. Those on hormone replacement therapy in the six months before enrollment, or who frequently (more than twice a week) took vitamin supplements or medications such as anti-inflammatories, analgesics, anti-allergic, or antidepressants were also considered ineligible for the study. Other exclusions included a history of cardiovascular disease, daily alcohol consumption exceeding 30 g, or a current smoking habit.

### 2.2. Biochemical Analysis

Fasting blood samples were centrifuged at 1600× *g* for 15 min to isolate serum or plasma. Samples were stored at −80 °C stored until analysis. With the exception of metabolic markers, all the HDL-accessory proteins were assessed by the spectrofluorometer Tecan Infinite M200 (Tecan Group Ltd., Männedorf, Switzerland).

#### 2.2.1. Metabolic Markers

Concentrations of glucose, insulin, total cholesterol, HDL-C, triglycerides, apolipoprotein A-1 (ApoA), and apolipoprotein B100 (ApoB) were measured at the Central Laboratory of S. Anna University Hospital in Ferrara using standard enzymatic colorimetric methods (Beckman Coulter, Brea, CA, USA). LDL cholesterol values were derived using Friedewald’s equation, while insulin resistance was assessed via the HOMA-IR index, calculated as follows:

$$HOMA-IR\;Index=\frac{fasting\;serum\;insulin\left(\frac{\mu U}{ml}\right)*fasting\;plasma\;glucose(\frac{mg}{dl})}{405}$$

#### 2.2.2. PON1-Arylesterase Activity

PON1-arylesterase activity was determined by combining 10 µL of 24-fold diluted serum with 240 µL of a reaction mixture containing 1 mmol/L phenylacetate and 0.9 mmol/L CaCl_2_ in 9 mmol/L Tris-HCl, pH 8 [16]. Enzyme activity was calculated using a molar extinction coefficient of 1.3 × 10^3^ L^−1^ mol^−1^ cm^−1^ (wavelength = 270 nm) and expressed in kilounits per liter (kU/L). One unit of activity signified the production of 1 mmol of phenol per minute under assay conditions. The intra-assay coefficient of variation (CV) was 3.8%, and the inter-assay CV was 9.7%.

#### 2.2.3. PON1-Lactonase Activity

The lactonase activity of PON1 was determined through enzymatic hydrolysis of dihydrocoumarin (DHC), a specific artificial substrate. Briefly, 3 µL of serum (diluted 1:5) were added in duplicate to the wells in a UV microplate (Sigma-Aldrich, St. Louis, MO, USA). Subsequently, each well was treated with 97 µL of Reaction Buffer (50 mM Tris, 1 mM CaCl_2_, pH 7.5) and 100 µL of dihydrocoumarin (Sigma-Aldrich, St. Louis, MO, USA, Cat. No. D104809) at the final concentration of 2 mM. The resulting absorbance was measured at the wavelength of 270 nm following a kinetic cycle every 30 s for 10 min at 37 °C. The absorbance was converted into enzyme units (U)/L of active enzyme using a molar extinction coefficient ε = 0.0136 µM^−1^ cm^−1^. The assay demonstrated good reproducibility, with an intra-assay coefficient of variation of 5.57% and an inter-assay coefficient of variation of 10.1%

#### 2.2.4. Lp-PLA2 Activity

For Lp-PLA2 assessment, we employed 2-thio PAF as the substrate [7]. The enzyme’s action on this substrate, specifically hydrolysis at the Sn^−2^ position, led to the formation of free thiols, which we then detected using Ellman’s procedure. We determined enzyme activity, expressed in U/L, by utilizing a molar extinction coefficient of 13.6 × 10^3^ L^−1^⋅mol^−1^⋅cm^−1^ at a wavelength of 410 nm. The assay demonstrated good reproducibility, with an intra-assay coefficient of variation of 4.8% and an inter-assay coefficient of variation of 10.1%.

#### 2.2.5. GPX3 Activity

The glutathione peroxidase (GPx) activity was determined through the oxidation of GSH to produce GSSG as part of the reaction in which it reduces cumene hydroperoxide [33]. Glutathione reductase (GR) then reduces the GSSG to produce GSH, and in the same reaction consumes NADPH. The decrease in NADPH (measured at OD = 340 nm) is proportional to GPx activity. In summary, 5 μL of serum (diluited 1:10) were dispensed in duplicate to the wells in a UV microplate. Then, 100 μL of mix A composed by 3 U/L Gluthatione reductase (Sigma-Aldrich, Cat. No. G3664-500UN), 1 mM L-Gluthatione reduced (Sigma-Aldrich, Cat. No. G4251) and PBS were added to each well. The plate was incubated for 10 min at 37 °C. Subsequently, 100 μL of mix B made of 0.2 mM NADPH (Sigma-Aldrich Cat. No. N1630), 0.4 mM tBH (Sigma-Aldrich Cat. No. 814006) and PBS were added to each well. Finally, the resulting absorbance was measured at the wavelength of 340 nm following a kinetic cycle every 30 s for 20 min at 37 °C. The assay showed good reproducibility, with an intra-assay coefficient of variation of 4.2% and an inter-assay coefficient of variation of 10%.

#### 2.2.6. MPO Activity

The MPO activity assay was conducted following the protocol established in our previous study [14]. In summary, 100 μL of anti-MPO polyclonal antibodies (Calbiochem, San Diego, CA, USA, Cat. No. 475915) were used to coat the wells of an ELISA microplate, and the plate was incubated overnight at 4 °C. After cycles of washings, the blocking (5% Bovine Serum Albumin) (BSA) 10 μL of serum (diluted 1:5) or standard (ranging from 0.39 to 25 ng/mL MPO, Calbiochem, Cat. No. 475911) were added in duplicate to the wells. The plate was then incubated for 1 h and each well was subsequently treated with H_2_O_2_ (final concentration of 196 μM) and 200 μM AmpliFlu Red (Sigma-Aldrich, Cat. No. 90101), both diluted in 20 mM citrate buffer (pH 6). The resulting fluorescence was measured at an excitation wavelength of 535 nm and an emission wavelength of 590 nm every 30 s for 10 min at 37 °C. The fluorescence readings were converted into enzyme units (U)/L of active enzyme using a standard curve, as previously described [34]. The assay showed intra- and inter-assay coefficients of variation of 5.8% and 10.4%, respectively, with a limit of detection (LoD) of 0.074 μU.

#### 2.2.7. Total MPO Protein Concentration

The concentration of total MPO was determined by a commercially available ELISA kit by following the manufacturer’s instructions, with some changes (Human MPO, Aviscera, Santa Clara, CA, USA, Cat. No. SK00172-09). The assay showed intra- and inter-assay coefficients of variation of 5.8% and 10.4%, respectively, with a limit of detection (LoD) of 0.074 μU. The amount of MPO was then calculated by interpolation with the standard curve.

#### 2.2.8. Oxidized HDL

This method is based on the enzyme horseradish peroxidase (HRP) and the fluorochrome AmpliFlu Red that can quantify the lipid peroxide content (without cholesterol oxidase) per mg of HDL-C [34]. A quality control from the pooled serum of all study samples was set up, combining 10 μL from each of them. Then, 40 μL of serum were precipitated with 40 μL of HDL Cholesterol precipitating Reagent (20% *w/v* polyethylene glycol in 0.1M glycine buffer at pH 10). All samples were centrifuged for 10 min at 2000× *g* at RT and the supernatant containing the HDL fraction was aspirated. The total amount of cholesterol was quantified using a commercial kit (Cholesterol Quantification Assay kit, Sigma-Aldrich Cat. No. CS0005). Both samples and reagents were prepared in Reaction Buffer 0.5M KH_2_PO_4_, 0.25M NaCl, 25 mM cholic acid, 0.5%Tryton X-100 at pH 7.4. Subsequently, 50 μL of the Reaction Buffer (negative control) and pooled control and samples (diluted 1:4) were dispensed in duplicate to the wells of a flat black bottom microplate. Then, 50 μL of 5 U/mL HRP (Sigma-Aldrich, Cat. No. P8375) were added to each well and the plate was incubated for 30 min at 37 °C. Finally, 0.3 mM AmpliFlu Red (Sigma-Aldrich, Cat. No. 90101) were added and the resulting fluorescence was measured at an excitation wavelength of 530 nm and an emission wavelength of 590 nm every minute for 1 h at 37 °C. The mean value of fluorescence units (arbitrary units) was subtracted from the blank by using the following equation:

*Fluorescence in HDL sample (FU)* − *Fluorescence in blank (no HDL) (FU)* = *HDLox_sample*

The resulting value of both the samples and the pooled serum was then normalized for Total Cholesterol amount (mg/dL) by using the following equation:

*HDLox_sample/Tot.Cholesterol mg/dL* = *HDLox_normalized*

The resulting value of each sample was standardized against the value of the pooled control by using the following equation:

*HDLox_normalized_samples/HDLox_normalized_pool* = *nHDLox*

The assay showed intra- and inter-assay coefficients of variation of 1.94% and 3.93%, respectively.

### 2.3. HDL-Antioxidant/Anti-Inflammatory Score (HDL-AAS)

To estimate the overall contribution of the assessed antioxidant and anti-inflammatory proteins of HDLs, we developed a composite score (HDL-antioxidant/anti-inflammatory score (HDL-AAS) that incorporates the relative contribution of each component of this biological parameter. Specifically, the contribution of each marker was determined based on its quartile distribution and its known protective or harmful effects. The schematic presentation of these scores is reported in Supplementary Table S1.

### 2.4. Nutritional Assessment

Nutritional assessment was conducted through two independent 24 h dietary recalls, a retrospective and quantitative method to gather information about foods and beverages consumed by the participant in the 24 h prior to the visit [35]. Two recalls were collected by trained interviewers, the first one in person on the day of the visit, and the second one after two months over the phone. Data from 24 h recall were analyzed using the nutrient analysis software Winfood^®^ PRO 3.9.x (Medimatica Surl, Teramo, Italy) to obtain total energy and macro and micronutrient intake for each individual interview. Results were the average of the two 24 h recalls.

Hydrophilic (H-ORAC) and lipophilic ORAC (L-ORAC) for water-soluble and fat-soluble antioxidants, respectively, and total ORAC values were also obtained from Winfood^®^ PRO 3.9.x.

### 2.5. Statistical Analysis

The Shapiro–Wilk test was used to evaluate the normality of the continuous variables in the study. Normally distributed variables are reported as mean ± standard deviation (SD), while non-normally distributed variables are expressed as median (interquartile range). Associations between variables of interest were assessed using Pearson’s correlation for normally distributed data and Spearman’s correlation for non-normally distributed data. When non-parametric variables became normal after being log10-transformed, Pearson’s test was used to assess the potential correlation.

Stepwise multiple regression analysis was then conducted with HDL functional score (HDL-AAS) as the outcome variable to verify the independence of associations identified in univariate analysis. Multicollinearity was assessed using the variance inflation factor (VIF), with values > 2.5 indicating exclusion of the affected variable. Variables were entered into the model if *p* ≤ 0.05 and removed if *p* > 0.10. A two-tailed *p*-value < 0.05 was considered statistically significant.

Data analysis was performed using SPSS Statistics for Windows, version 26.0 (SPSS, Inc., Chicago, IL, USA), and a *p* < 0.05 was considered as statistically significant.

## 3. Results

### Main Characteristics of the Study Population

The study involved 145 older women aged 52 years (mean) of whom 74 were postmenopausal. Prevalence of medical conditions, such as dyslipidemia, hypertension, and obesity was lower than 20%, with no subject with current or a history of cardiovascular disease (CVD (Table 1). Table 2 shows the results of macro-and micro-nutrients assessment, with a particular focus on the intake of different types of FAs, antioxidants and nutrients involved in regulating redox balance.

The comparison between levels of selected HDL-accessory proteins and menopausal status revealed no significant differences or discernible trend indicating a reduction in protective components or an increase in harmful constituents of this lipoprotein.

Given the potential role of diet in shaping HDL functionality, the relationship between the HDL accessory proteins (assessed as enzymatic activity or protein concentration) and FA/antioxidant intake (Table 3) was investigated. Among the examined markers, MPO activity, concentration, and -specific activity showed the highest number of significant correlations with dietary factors (Table 3). Specifically, MPO activity was inversely correlated with PUFA, omega-3, and omega-6 intake (*p* < 0.05 for all) (Table 3). MPO activity also negatively correlated with polyphenols (*p* < 0.001) and all three ORAC scores (*p* < 0.05) (Table 3). MPO concentration showed similar correlation with these dietary antioxidant scores (with the exception of L-ORAC).

The ratio between the two aforementioned measures, i.e., MPO-specific activity, was negatively associated with omega-3 (*p* < 0.001), omega-6 (*p* < 0.05), and vitamin A, E, and C, and polyphenols (*p* < 0.05 for all). The other potential harmful HDL-associated enzyme, Lp-PLA2, was also negatively related with antioxidant intake, but those collectively detected (H-ORAC and Total-ORAC, *p* < 0.05 for all).

Conversely, higher dietary intake of omega-3 and all ORAC scores were positively associated with serum levels of antioxidant and potentially protective Gpx3.

Further detected correlations were the following: L-CAT, positively, with polyphenols (*p* < 0.05); APOA1, positively, with omega-3 and its ratio to total PUFAs (*p* < 0.05); and PON1-arylesterase negatively with vitamin A (*p* < 0.05).

**Figure 1:**
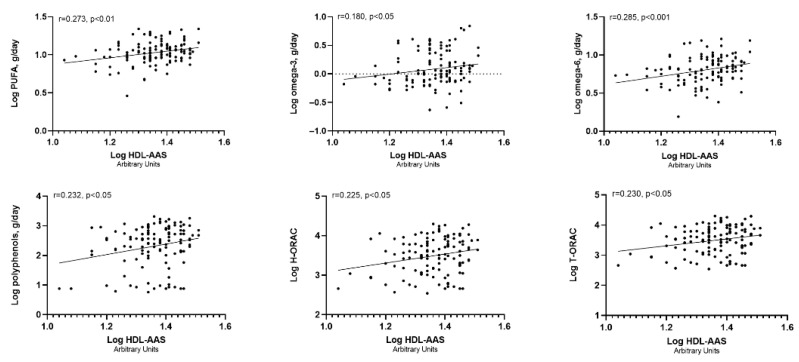
Box plot displaying the relationship between the composite HDL-AAS score and key nutrients or antioxidant potential of the diet (from the upper left to the bottom right: PUFAs, omega-3, omega-6, polyphenols, H-ORAC, and T-ORAC).

After identifying the nutrients that displayed a significative relationship with individual serum markers, we extended our analysis to assess whether these nutrients were also associated with the composite HDL-AAS score (Figure 1). HDL-AAS was positively related to several key dietary components and indicators of antioxidant potential, including PUFAs, omega-3, omega-6, polyphenols, hydrophilic H-ORAC, and T-ORAC. All associations reached statistical significance (*p* < 0.05), with the strongest correlation being observed for PUFAs (*p* < 0.001). Given the relevance of PUFAs, we further explored the relationship between individual fatty acids within this group and HDL-AAS. Among arachidonic acid, eicosapentaenoic acid, docosahexaenoic acid, linoleic acid, and linolenic acid (LA), only LA showed a significant association with the score (r = 0.245, *p* < 0.001). Supplementary Figure S1 illustrates representative scatter plots comparing HDL-AAS with LA and arachidonic acid.

Next, we aimed at evaluating if these associations held true even after accounting for potential confounding factors such as age, menopause, obesity, HOMA-IR index, physical activity, dyslipidemia, and hypertension (Table 4). We found that all the previously identified nutrients and indicators of the antioxidant potential of the diet—PUFA, omega-3, omega-6, polyphenols, H-ORAC, and total T-ORAC—remained significantly linked with HDL-AAS, even after adjusting for the aforementioned confounders. Our analysis revealed that omega-6 and PUFA had the strongest association with HDL-AAS (*p* < 0.001 for both β coefficients), consistent with initial correlation data. These two variables were the only independent predictors of HDL-AAS when entered into separate multiple regression models, alongside omega-3 (only in the model with omega-3, to avoid collinearity with PUFA), polyphenols, H-ORAC, or T-ORAC.

**Figure 2:**
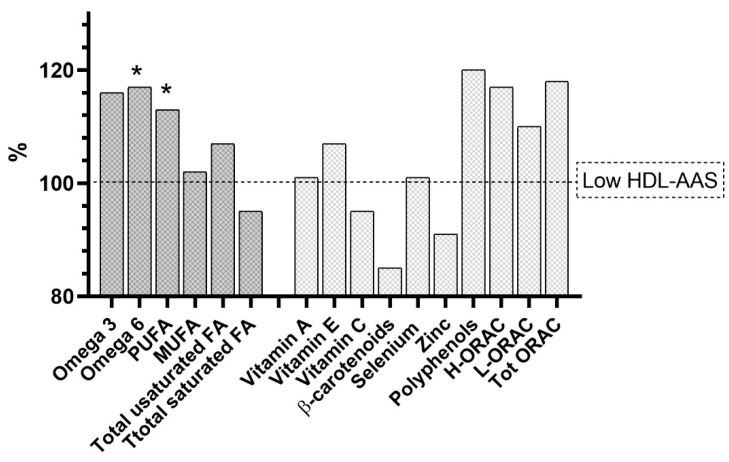
Intake of dietary FAs and antioxidants in individuals with high HDL-AAS to those with low HDL-AAS (high/low was based on median values). * *p* < 0.05, for the comparison between women with high HDL-AAS compared to those with low HDL-AAS.

The outcomes of the multivariate analysis suggest a robust relationship between these nutrients and HDL-AAS, independent of common health factors. To better understand how HDL-AAS relates to dietary FAs and antioxidants, we compared nutrient intake in individuals with high HDL-AAS to those with low HDL-AAS (based on median values, Figure 2). As shown in Figure 2, individuals with higher HDL-AAS scores consumed more omega-3 and omega-6, with an increase of at least 15% compared with those with a low HDL-AAS score (only the difference of the former was statistically significant). While the rise in PUFA intake was slightly less pronounced, it was still significant, mirroring the trend seen with omega-6 (most likely because these FAs represent the majority of the PUFAs of the subject’s diet). Other FAs, such as saturated FAs, showed only moderate or negligible changes. Regarding antioxidants, polyphenols, H-ORAC, and T-ORAC exhibited the most notable increases, at 20%, 17%, and 18%, respectively. Other antioxidants showed only negligible or no increase.

## 4. Discussion

This study demonstrates that dietary intake of PUFAs, including both omega-3 and omega-6, along with overall antioxidant content, is associated, although weakly, with an improved antioxidant and anti-inflammatory functional capacity of HDLs. These effects were particularly evident when examining specific HDL-associated proteins, such as MPO and Gpx-3, as well as through a composite score reflecting overall HDL function (HDL-AAS). However, no significant associations were found between PON1 enzymatic activities or oxidized HDL levels and any of the considered nutrients.

Despite increasing interest, research on how diet influences HDL-associated proteins remains limited and often generated conflicting results. Among these proteins, PON1 has been the most extensively studied [36]. Along with APOA1, PON1 plays a crucial role in HDL’s antioxidant function, particularly by neutralizing lipid peroxides on LDL particles and macrophage membranes [37,38,39,40,41]. However, the majority of the evidence supporting the beneficial role of these proteins comes from in vitro studies or small-scale dietary interventions, and promising preclinical findings have not been consistently translated into clinical outcomes [37,38,39].

For instance, while antioxidant-rich extracts and vitamins have been shown to enhance PON1 expression and activity in cell and animal models [42], population-based studies report inconsistent results [43,44]. The Mediterranean diet, extra virgin olive oil consumption, and dietary shifts to increase intake of omega-3, omega-6, and antioxidants have yielded mixed outcomes, ranging from no detectable effect to modest improvements in PON1 activity [19,36]. These discrepancies may stem from methodological differences in assessing PON1, whether through protein concentration or its distinct enzymatic activities (lactonase, arylesterase, paraoxonase, thiolactonase, and diazoxonase), which often produce non-overlapping results [45,46].

In this study, we did not measure PON1 concentration directly; instead, we evaluated two of its key enzymatic activities, arylesterase and lactonase. On the one hand, we focused on arylesterase activity because it is a widely used and reliable measure that correlates closely with the overall concentration of PON1 protein. On the other hand, lactonase activity is considered to be PON1’s main physiological role, responsible for breaking down lipolactones [47]. These molecules are formed from the oxidation of lipids, particularly those contained within LDL, and are regarded as the enzyme’s natural, endogenous substrates [47].

Interestingly, we did not find a correlation between PUFA intake and PON1 activity. This is likely attributable to the different effects of PUFAs on redox balance. Indeed, while these dietary components may enhance the expression of the gene of PON1 as well as other antioxidant proteins [48,49], they are also highly prone to oxidative degradation, whether spontaneous or chemically induced, due to the presence of electron-rich double bonds in their structure [50,51]. The resulting increase in ROS production and lipid peroxidation by-products may, in turn, inhibit the enzyme’s activity (as widely demonstrated in in vitro studies [52]), canceling out any potential benefit from increased expression.

Two additional HDL-related antioxidant proteins examined in this study were lecithin-cholesterol acyltransferase (L-CAT) and, notably, Gpx-3. L-CAT primarily facilitates the conversion of unesterified cholesterol into more hydrophobic esterified cholesterol, promoting the remodeling of small preβ particles into spherical particles and facilitating transfer of cholesterol from peripheral cells to HDL [53,54]. The observed positive correlation between L-CAT activity and omega-3 intake aligns with prior findings on PUFA’s beneficial effects [26,27].

Gpx-3, part of the glutathione peroxidase family, is among the body’s most powerful endogenous antioxidant enzymes, alongside superoxide dismutase and catalase [55,56]. The selective link we identified between omega-3 intake and Gpx-3 activity is novel in human studies. This association may be explained by omega-3’s role as a ligand for PPAR nuclear receptors, which appear to upregulate Gpx-3 expression [57]. The influence of dietary antioxidants on Gpx-3 was also notable, likely due to their established role in modulating redox-sensitive enzymes [56,58,59].

Our findings on lipoprotein-associated phospholipase A2 (LP-PLA2) are broadly consistent with the existing, albeit limited, literature. LP-PLA2 is now recognized as a positive biomarker for coronary heart disease (CHD), as its enzymatic activity generates pro-inflammatory and pro-oxidant metabolites [7,60,61,62]. Similarly to prior studies using mixed antioxidants, we observed that higher antioxidant intake correlated with reduced LP-PLA2 activity [28,29].

A comparable trend was observed for MPO, a heme peroxidase abundant in neutrophils and macrophages that plays a key role in innate immunity and inflammation. MPO produces hypochlorous acid (HOCl), which—while antimicrobial—can also contribute to tissue damage in chronic inflammation [14,63,64]. The inverse correlation between omega-3 intake and MPO activity aligns with in vitro studies [22] and an animal study showing that fish oil reduces MPO activity [65]. Conversely, Anderson et al. reported no change in total MPO concentration in healthy individuals after marine omega-3 supplementation [30].

Finally, the observed reduction in MPO activity with higher antioxidant intake is supported by extensive preclinical evidence. Vitamins C and E, carotenoids, and other antioxidants have been shown to inhibit MPO’s pro-oxidant effects [66,67]. These findings are further corroborated by human studies demonstrating decreased MPO levels with increased fruit and vegetable consumption [68].

The novelty of the data reported herein is the concept of a comprehensive HDL-AAS. This score uniquely combines the beneficial (anti-inflammatory and antioxidant) and harmful (pro-oxidant and pro-inflammatory) effects of various proteins on HDL. Interestingly, we observed that higher consumption of all dietary fatty acids (total PUFAs, omega-3, and omega-6) and antioxidants (polyphenols, H-ORAC, and T-ORAC), individually or together, was linked to an improved HDL-AAS.

It is not surprising that most single antioxidants, except for polyphenols, fail to enhance HDL’s protective properties. A broad consensus now supports the notion that low-weight antioxidants are ineffective alone in vivo [69,70]; they require synergy with each other and endogenous enzymatic systems [69,70,71]. This is exemplified by vitamins C and E. While being very effective in vitro, many antioxidants can become prooxidants or lose efficacy in vivo [69,70,71]. Vitamin C plays a pivotal role in regenerating vitamin E (α-tocopherol) from its radical form through chemical reduction [69,70,71]. The capacity of ascorbic acid to sustain the antioxidant activity of α-tocopherol within the phospholipid bilayer is closely dependent on the efficiency of endogenous antioxidant systems. These systems, particularly those relying on glutathione and NAD(P)H, are essential for maintaining adequate intracellular levels of ascorbic acid, thereby enabling continued regeneration of α-tocopherol [72]. Without this partnership, vitamin E, the primary antioxidant in LDL and cell membranes, could paradoxically promote oxidative damage [70]. This explains why epidemiological studies have found no definitive protective benefit of vitamin E or other dietary antioxidant supplementation in diseases involving oxidative stress [73,74,75,76,77,78].

Notably, all the associations found with HDL-AAS held true, even when considering other factors like age, menopause, existing health conditions, and lifestyle. Interestingly, among all these dietary components, total PUFAs, driven by omega-6, were the strongest independent predictors of HDL-AAS. This finding aligns with several reports indicating that linoleic acid (LA), a polyunsaturated FA (PUFA), can replace saturated FAs in HDL, thereby boosting its levels, stability levels, and function [79,80]. However, some studies have not shown significant improvements in these parameters [81].

The seemingly unexpected positive correlation between the putatively pro-inflammatory omega-6 fatty acids and the HDL-related score (HDL-AAS), which is also influenced by the inverse association with MPO activity (Table 3), can be interpreted in two ways. First, it is important to consider that, although omega-6 fatty acids are known to be precursors of pro-inflammatory eicosanoids, the impact of these fatty acids on inflammatory responses is not exclusively dependent on their intake. Instead, a more prominent role in regulating the balance between pro- and anti-inflammatory responses is played by the omega-6/omega-3 ratio. In our cohort, the relatively low ratio (6.2) may help explain the observed discrepancy. Second, the biological role and health impact of omega-6 fatty acids remain incompletely understood and are still under active investigation [82].

The potentially adverse effects of omega-6 FAs are primarily attributed to their role as precursors of pro-inflammatory eicosanoids [83]. However, emerging evidence suggests that omega-6 FAs, in particular LA (which showed a positive correlation with HDL-AAS) may exert beneficial, or at least neutral, effects under certain conditions. In support of this paradigm, Kammerer et al. demonstrated that 13-HODE, a hydroxylated derivative of LA [84], enhances cholesterol efflux from macrophages, thereby improving HDL-mediated reverse cholesterol transport [85]. Similarly, Vaisar et al. found that conjugated LA boosts HDL’s anti-inflammatory properties and promotes favorable cholesterol efflux in obese mice [86]. This body of evidence has been reinforced by recent large-scale meta-analyses. Notably, Marklund et al. analyzed data from 30 cohort studies and found that higher circulating and tissue levels of LA and arachidonic acid were associated with a reduced risk of major cardiovascular events [87]. Consistently, a meta-analysis encompassing 150 studies concluded that higher dietary intake and circulating levels of omega-6 fatty acids are predominantly protective, reducing the risk of cardiovascular diseases, certain cancers, and all-cause mortality in the general population [88]. Together, these findings, along with other evidence, challenge the notion that omega-6 FAs are uniformly pro-atherogenic and pro-inflammatory.

This study has several limitations. Firstly, the HDL-AAS score does not include an assessment of macrophage cholesterol efflux and, more broadly, does not fully capture the entire spectrum of HDL functionality. However, the score was developed with two primary objectives: (1) to evaluate the main contributors to two key properties of HDL, its antioxidant and anti-inflammatory capacities, which are known to play a substantial role in the overall biological activity of the lipoprotein [6,89]; and (2) to incorporate readily measurable markers, thereby facilitating the score’s application in both research and clinical settings through a streamlined and efficient evaluation process. Considering these points, the absence of Serum Amyloid A (SAA) assessment represents a limitation of the HDL-AAS score. SAA is known to displace key HDL-associated proteins such as PON1 and APOA1, thereby impairing HDL’s antioxidant capacity, particularly under conditions of elevated systemic inflammation [90]. This omission may affect the reliability and completeness of the HDL-AAS score. Secondly, we acknowledge that the HDL-AAS score requires both biological and clinical validation. For this reason, future studies will be specifically designed to address this need and strengthen the reliability and applicability of the score. Thirdly, our sample size may have been insufficient to reliably detect small effects, and for this reason, our non-significant findings should be interpreted with caution. Fourthly, relying on 24 h dietary recalls for nutritional assessment presents notable limitations. These methods are often prone to inaccuracies due to their dependence on self-reporting, which can result in misreporting of food intake and incorrect estimation of nutrient and energy intake. Moreover, two single-day recalls may not accurately reflect an individual’s habitual dietary patterns. Additionally, a 24 h recall, contrary to a nutritional intervention trial, does not allow us to identify a direct causal relationship between the intake of specific nutrients and the outcome of interest. Incorporating direct measurements of FAs and antioxidant levels in the participants’ blood could have enhanced the reliability and validity of the dietary data. Finally, it must be emphasized that since our study was conducted only on women, the findings cannot be completely generalized to the broader population.

## 5. Conclusions

To the best of our knowledge, this is one of the first studies to demonstrate that PUFAs, particularly omega-6, along with total dietary antioxidants, may enhance the protective properties of HDLs in women. Further research involving a larger sample size and employing more accurate methods for assessing dietary intake is necessary to confirm and expand upon these findings.

## Figures and Tables

**Table 1:** Principal characteristics of the sample.

Variables	
Number	145
Age, years	52 ± 7
Postmenopausal status, n (%)	74 (50)
BMI, Kg/m^2^	25 (23–28)
- Comorbidities and lifestyle	
Hypertension, n (%)	22, (15)
Obesity, n (%)	20, (14)
Dyslipidemia, n (%)	9 (6)
Physical activity, n (%)	35 (24)
Smoking, n (%)	10 (7)
- Metabolic markers	
HDL-C, mg/dL	66 ± 14
LDL-C, mg/dL	148 ± 33
Triglycerides, mg/dL	76 (60–103)
Total Cholesterol, mg/dL	231 ± 37
ApoA1, mg/dL	176 ± 33
ApoB, mg/dL	92 ± 18
Glucose, mg/dL	94 (90–100)
Insulin, mg/dL	5 (3–8)
HOMA-IR index	1.2 (0.8–1.8)
- Markers of HDL function	
PON1-arylesterase, U/L	92 ± 20
PON1-Lactonase activity, U/L	85 ± 19
Gpx3, U/L	183 ± 28
Lp-PLA2, U/L	12.8 ± 2.9
L-CAT, arbitrary units	1.5 (1.4–1.6)
Ox-HDL, mg/dL	1.0 (0.9–1.2)
MPO-concentration, mg/dL	0.77 (0.59–1.29)
MPO-activity, U/L	0.01 (0.01–0.03)
MPO-specific activity, U/µg	0.15 (0.10–0.25)

Variables are expressed as mean ± SD or median (interquartile range) when normally and non-normally distributed, respectively. Abbreviations: BMI, body mass index; HDL-C, High density lipoprotein-cholesterol; LDL-C, Low density lipoprotein-cholesterol; ApoA1, apolipoprotein A1, PON1, paraoxoanse-1; Gpx3, glutathione peroxidase 3; Lp-PLA2, lipoprotein-phospholipase A2, L-CAT, lecithin cholesterol acyltransferase; MPO, myeloperoxid.

**Table 2:** Nutrients intake.

Nutrients	
Calories, Kcal/day	1993 ± 391
Proteins, g/day	86 ± 17
Lipids, g/day	89 ± 19
Fiber, g/day	19 ± 5
- FA	
Saturated FA, g/day	22 (17–27)
Unsaturated FA, g/day	6.5 (3.4–8.5)
Saturated FA/unsaturated FA	2.5 (2.0–3.0)
MUFA, g/day	0.02 ± 0.004
PUFA, g/day	8.5 (7–11)
Omega-3, g/day	1.0 (0.7–1.7)
Omega-6, g/day	6.0 (5.0–8.0)
Omega-6/omega-3	4.6 (3.8–6.8)
- Antioxidants	
Vitamin A, µg/day	1181 (867–1444)
Vitamin E, mg/day	15 (12–17)
Vitamin C, µg/day	183 (128–227)
B-carotenoid, mg/day	134 (49–520)
Selenium, µg/day	31 (22–51)
Zinc, µg/day	11 (8–13)
Polyphenols, mg/day	428 ± 189
H-ORAC, µmoles/day	3204 (1162–6250)
L-ORAC, µmoles/day	68 (15–154)
Total ORAC, µmoles/day	3235 (1183–6238)

Variables are expressed as mean ± SD or median (interquartile range) when normally and non-normally distributed, respectively. Abbreviations: FA, fatty acid, MUFA, monounsaturated; PUFA, polyunsaturated.

**Table 3:** Correlation coefficients between HDL-accessory proteins and dietary FAs or antioxidants.

	Lp-PLA2 Activity	PON1 Arylesterase Activity	PON1 Lactonase Activity	MPO Activity	MPO Concentration	MPO-Specific Activity	GPx3 Activity	LCAT Activity	oxHDL	APOA1
Saturated FA	0.035	−0.075	−0.117	−0.076	* 0.156	−0.112	−0.031	−0.028	0.1	0.055
PUFA	0.056	0.035	−0.1	−0.184 *	−0.123	−0.288	0.073	−0.06	0.034	0.123
MUFA	0.069	−0.079	−0.177	0.046	−0.121	−0.118	0.111	−0.22	0.004	0.075
Unsaturated FA tot	−0.021	0.013	0.011	0.01	−0.058	−0.089	0.165	−0.015	0.113	−0.035
Saturated/unsaturated	0.035	−0.041	−0.056	−0.04	0.023	0.05	−0.166	0.005	−0.08	0.055
Omega−3	−0.03	−0.111	−0.128	−0.158 *	−0.105	−0.265 **	0.219 *	−0.036	0.063	0.088
Omega−6	0.046	0.108	−0.171 *	−0.212 *	−0.129	−0.224 *	−0.042	−0.003	−0.088	0.247 *
Omega−6/omega−3	0.059	0.189	0.154	−0.018	−0.08	0.096	−0.145	−0.002	−0.122	0.05
Alpha-Tocopherol	−0.034	0.025	−0.031	0.028	−0.121	−0.190 *	0.196	0.181	0.049	−0.06
Vitamin A	−0.057	−0.195 *	−0.12	0.091	−0.122	−0.186 *	0.082	−0.12	0.16	−0.034
Vitamin C	0.05	−0.096	−0.099	0.058	−0.109	−0.175 *	0.119	−0.063	0.144	0.091
Beta-cartotene	−0.081	−0.067	−0.025	−0.077	−0.123	−0.094	0.123	0.186 *	0.162	−0.099
Polyphenols	−0.141	0.101	0.149	−0.218 **	−0.192 *	−0.170 *	0.081	0.068	0.02	0.114
Selenium	−0.033	−0.092	−0.06	0.129	−0.123	−0.101	0.175	−0.037	0.086	−0.025
H-ORAC	−0.253 *	0.086	0.151	−0.233 *	−0.187 *	−0.173	0.203 *	0.002	0.027	0.116
L-ORAC	−0.064	0.038	−0.045	−0.218 *	−0.150	−0.187	0.238 *	−0.027	0.092	0.046
Total-ORAC	−0.249 *	0.084	0.151	−0.235 *	−0.188 *	−0.175	0.206 *	0.004	0.028	0.115

* *p* < 0.05; ** *p* < 0.001. Abbreviations: PON1, paraoxoanse-1; Gpx3, glutathione peroxidase 3; Lp-PLA2, lipoprotein-phospholipase A2, L-CAT, lecithin cholesterol acyltransferase; MPO, myeloperoxidase, SFA, saturated fatty acid, MUFA, monounsaturated; PUFA, polyunsaturated.

**Table 4:** Independent association between HDL functional score (HDL-AAS) and selected FA or antioxidants as assessed by stepwise multiple regression analysis.

Explanatory Variables	Unstandardized Coefficients	Standard Error	Standardized Coefficients (β)	Adjusted R^2 #^
PUFA	8.94	2.48	0.289 ***	0.204 ***
Omega-3	3.136	1.284	0.201 **	0.160 *
Omega-6	6.895	2.097	0.267 ***	0.191 **
Polyphenols	1.352	0.532	0.212 *	0.179 **
H-ORAC	2.121	0.825	0.215 *	0.160 *
T-ORAC	2.164	0.828	0.220 *	0.161 *

All multiple regression models include age, menopausal status, HOMA-IR index, obesity, hypertension, dyslipidemia, smoking, and physical activity. ^#^ Among the included covariates, only age and obesity remained significantly associated with HDL-AAS (all models). * *p* < 0.05; ** *p* < 0.01; *** *p* < 0.001. Abbreviations: PUFAs, polyunsaturated fatty acids.

## Data Availability

Data will be available upon reasonable request.

## Supplementary Materials

The following supporting information can be downloaded at https://www.mdpi.com/article/10.3390/antiox14101221/s1, Figure S1: Assessment of HDL-antioxidant/anti-inflammatory score (HDL-AAS): points added according to marker quartile; Table S1: Scatter plot for the correlation between HDL-ASS and linolenic acid, and Arachidonic acid.
