# Supplementary material for: Effect of Dietary PUFAs and Antioxidants on Antioxidant and Anti-Inflammatory Functions of HDL in a Cohort of Women
Source: Antioxidants (Basel). 2025 Oct 10;14(10):1221. doi: 10.3390/antiox14101221 (PMC12561721; doi:10.3390/antiox14101221)
Supplement: Supplementary file 1 [file antioxidants-14-01221-s001.zip › antioxidants-3861140-supplementary.pdf]

Supplementary Table S1: Assessment of HDL-antioxidant/anti-inflammatory score (HDL-AAS): points added according to marker quartile

Accessory Proteins	Quartile I	Quartile II	Quartile III	Quartile IV
**PON1-arylesterase**	1	2	3	**4**
**PON1-Lactonase**	1	2	3	**4**
**ApoA1**	1	2	3	**4**
**Lp-PLA2**	**4**	3	2	1
**L-CAT**	1	2	3	**4**
**Ox-HDL**	**4**	3	2	1
**MPO-Specific activity, U/L**	**4**	3	2	1

The HDL-AAS score is calculated by adding up individual scores for each protein, with higher scores given to proteins with beneficial biological activity and lower scores for those with harmful activity. Specifically, a protein's score is determined by its quartile: the highest scores are in quartile I for beneficial proteins, and the lowest scores are in quartile I for harmful proteins. We included MPO-specific activity in the HDL-FS assessment, but not MPO activity or MPO concentration, because they are already accounted for in other measurements and are therefore collinear.

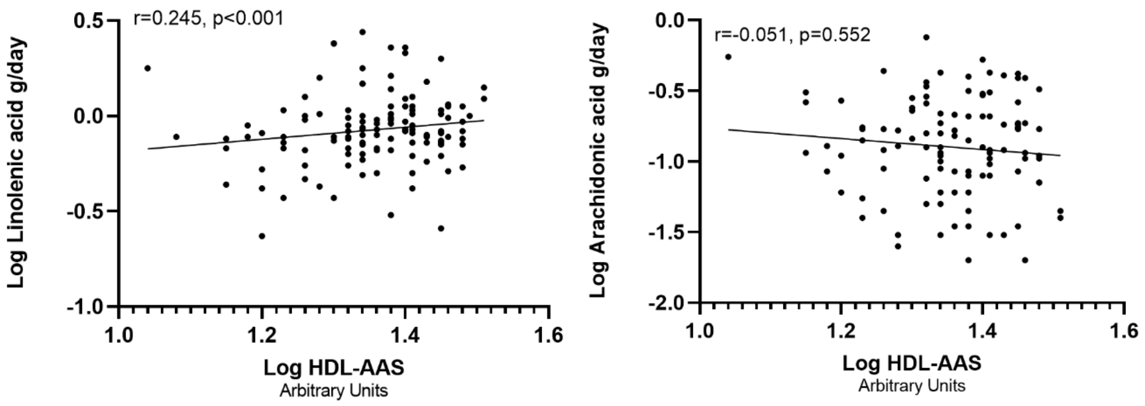

Supplementary Figure S1: Scatter plot for the correlation between HDL-ASS and linolenic acid, and Arachidonic acid
